# Lumbar Spinal Stenosis Has a Negative Impact on Quality of Life Compared with Other Comorbidities: An Epidemiological Cross-Sectional Study of 1862 Community-Dwelling Individuals

**DOI:** 10.1155/2013/590652

**Published:** 2013-12-23

**Authors:** Koji Otani, Shinichi Kikuchi, Shoji Yabuki, Tamaki Igarashi, Takuya Nikaido, Kazuyuki Watanabe, Shinichi Konno

**Affiliations:** Department of Orthopedic Surgery, Fukushima Medical University School of Medicine, 1 Hikarigaoka, Fukushima City 960-1295, Japan

## Abstract

Lumbar spinal stenosis (LSS) is common in the elderly. However, there have been few reports on its impact on quality of life (QoL) in community-dwelling individuals. The purpose of this study was to clarify how symptomatic LSS affects QoL at the community level. A total of 1862 people (697 males and 1165 females, most subjects were between 40 and 85 y.o.) agreed to participate and were interviewed. The presence of symptomatic LSS was assessed by a specially designed questionnaire. The Medical Outcomes Study 36-Item Short Form Health Survey (SF-36) was also administered. In addition, the presence of comorbid conditions that affect QoL, such as osteoarthritis of the knee and hip, cardiovascular disease, cerebrovascular disease, or respiratory disease, was also analyzed. The prevalence of symptomatic LSS gradually increased with age. Furthermore, the presence of symptomatic LSS had a strong negative effect on all 8 physical and mental domains and the physical component summary (PCS) (OR: 1.547–2.544) but not the mental component summary (MCS). In comparison with comorbid conditions, LSS had a much stronger negative impact on health-related QoL (HR-QoL). The current study confirmed that the presence of symptomatic LSS might have a strong negative influence on HR-QoL in the community setting.

## 1. Introduction

Radiographic lumbar spinal stenosis (LSS) is defined by the anteroposterior diameter of the spinal canal, lateral recesses, and neural foramina. Congenital (developmental) or acquired stenosis occurs as the space surrounding the neural tissues becomes smaller and eventually the tissues are compressed by a bulging disc (disc degeneration), facet osteoarthritis (facet OA), hypertrophy of the ligamentum flavum, or degenerative spondylolisthesis [1–5]. It is well known that radiographic changes do not always correlate with symptoms [6, 7]. Radiographic LSS commonly occurs in the elderly; however, the exact prevalence of symptomatic LSS has not been well defined [8–10].

One of the challenges in the epidemiological study of LSS is the lack of a gold standard to diagnose LSS. In the clinical setting, LSS is usually diagnosed based on subjective symptoms, such as pain and/or numbness of the lower extremity (or extremities) that worsens with ambulation, and physical findings supported by radiographic evidence of a narrow spinal canal [6, 7, 11, 12]. Recently, a diagnostic support tool for LSS (LSS-DST) has become available, which is a self-administered, self-reported history questionnaire consisting of 10 yes/no questions. This tool has been analyzed in derivation and validation studies and confirmed to have high sensitivity, specificity, and reproducibility [13]. Thus, it is considered to be useful for epidemiological studies of LSS.

Lumbar degenerative disorders including LSS are well known to impair general health-related quality of life (HR-QoL) and disease-specific outcome measures in clinic/hospital-based patients [14–21]. These patients are thought to show relatively severe symptoms compared to those of community-dwelling individuals, and there have been few studies regarding the influence of LSS on QoL in these individuals. In addition, there have been few reports on comparison of the impact on QoL between symptomatic LSS and comorbid conditions such as osteoarthritis of the knee and hip, cardiovascular disease, cerebrovascular disease, and respiratory disease. The purpose of the present study was to clarify the prevalence of symptomatic LSS in the community using a diagnostic support tool and to determine how symptomatic LSS affects QoL at the community level.

## 2. Methods

A total of 1862 people (697 males and 1165 females) agreed to participate in this study and were interviewed in 2004 when a public health survey was conducted by the local government. Approximately 60% of the public health survey participants comprised the total number of subjects in the present study, with written and informed consent. Their age ranged from 19 to 93 years old, with most subjects being between 40 and 85 years of age. The participants comprised approximately 21.5% of the local population (Table 1) of Tadami town, Tateiwa village, and Ina village in Fukushima prefecture, Japan. Ninety-three percent of this region is mountainous, and in 2004, 33.8% of the local residents were over the age of 65. All participants lived in their own houses, took care of themselves, and were able to walk independently. They had no experience of undergoing spine or brain surgery.

### 2.1. Definition of Symptomatic Lumbar Spinal Stenosis (LSS)

The presence of symptomatic lumbar spinal stenosis (LSS) was assessed by a validated diagnostic support tool, LSS-DST, consisting of 10 yes/no questions (Appendix). The sensitivity and specificity of LSS-DST had been confirmed to be 0.855 in the derivation data and 0.843 in the validation data and 0.791 and 0.781, respectively. The area under the receiver operating characteristic curve was 0.782 [13]. These results show that this tool is reliable for the diagnosis of symptomatic LSS and should be useful for epidemiological studies. In LSS-DST, those who answered “Yes” to questions (Qs)1–4 were judged as LSS positive. Those who answered “Yes” to one or more from Qs 1–4 and “Yes” to two or more from Qs 5–10 were also judged as LSS positive [13].

### 2.2. Evaluation of Quality of Life (QoL)

The Medical Outcomes Study 36-Item Short Form Health Survey (SF-36) (Japanese version 1.2) was used to evaluate health-related quality of life (HR-QoL) [22–24]. A Japanese version of SF-36 is available for both original and norm-based scores. (20–79, y.o., male/female). In this study, not only the original scores but also the standardized scores of the SF-36 were used because the original scores vary considerably with age and gender. In the standardized scores, the average score and one-standard deviation were 50 and 10, respectively, and a score of more than 50 points meant better QoL compared to the average Japanese person. Scores of all eight physical/mental domains, the physical component summary (PCS) and mental component summary (MCS) included in SF-36 were compared to those of norm-based scores (20–79, y.o., male/female) [24].

### 2.3. Comorbid Conditions

Musculoskeletal disorders such as osteoarthritis of the hip and the knee (hip OA, knee OA), cardiovascular disease, cerebrovascular disease, and respiratory disease may also impact HR-QoL in the elderly [25–28]. Ten experienced orthopedic surgeons judged the existence of hip and knee OA using Altman's criteria [29, 30]. In this study, in order to achieve better accuracy, one investigator (K.O.) instructed all physicians on how to judge the presence of hip and knee OA by Altman's criteria before assessment. The numbers of subjects who fulfilled all the evaluation criteria were 1750 for knee OA and 1862 for hip OA (age ranged from 19 to 93). Subsequently, six well-educated and experienced public health nurses asked all participants if they had received treatment for hypertension, cardiovascular disease, cerebrovascular disease, respiratory disease, or diabetes mellitus. In addition, all participants were assessed for their smoking status, and their pack years were calculated. The number of subjects who answered the entire questionnaire was 1710 (aged between 19 and 93), and they were asked whether they were undertaking treatment at the present time.

### 2.4. Inclusion/Exclusion Criteria

Participants who had had cerebral infarction or bleeding history without operation and could walk independently were included in this study. On the other hand, those who were unable to walk independently or to fill out the questionnaire due to eye problems and so forth or had had operations of the brain, spine, or fracture of lower extremity(-ies) within the previous year were excluded from this study.

### 2.5. Statistical Analysis

The chi-square test was used to assess the statistical differences of the prevalence of symptomatic LSS between genders in each age group, as shown in Table 2. A *t*-test was used to determine by which QoL was more affected, symptomatic LSS or any of comorbid conditions. A *P* value <0.05 was considered statistically significant.

In order to control the confounding variables, a multiple logistic regression analysis was performed for each HR-QoL measurement (all eight physical/mental domains and two summary scores of the SF-36) using 10 items in SF-36 as dependent variables and LSS and the other factors (BMI, smoking exposure, knee OA, hip OA, hypertension, cerebrovascular disease, respiratory disease, cardiovascular disease, and diabetic mellitus) as independent variables. The dependent variables were classified into two groups: standardized score ≥50 as better QoL and <50 as lower QoL compared with the national norm. For the independent variables, BMI and smoking exposure were used as continuous variables, and knee OA, hip OA, hypertension, cerebrovascular disease, respiratory disease, cardiovascular disease, and diabetic mellitus were used as nominal variables (0: none, 1: positive). The odds ratios with 95 percent confidence intervals (95% CI) for these factors were obtained for HR-QoL measurement (all eight physical/mental domains and two summary scores of SF-36). The degree of influence of each factor on QoL was judged by the odds ratio. This analysis was performed for those participants aged from 20 to 79 years old because the standardized (deviation) score of the national norm (20–79 y.o., male/female) was used.

All statistical analyses were performed using the STAT View software package (version 5.0, SAS Institute Inc., Cary, NC, USA).

### 2.6. Ethical Approval

This study was approved by the Ethical Committee of Fukushima Medical University.

## 3. Results

### 3.1. Prevalence of Lumbar Spinal Stenosis (LSS) (Table 2)

The prevalence of symptomatic lumbar spinal stenosis (LSS) gradually increased with age. In subjects younger than 50 years old, the prevalence was less than 10%. In the 55–64 age group, approximately 15% of subjects were judged to be symptomatic LSS positive. Twenty percent of males and females aged 65–69 were judged to be symptomatic LSS positive. In subjects older than 70 years old, the prevalence of symptomatic LSS in females increased further, up to 45-50%, whereas that in males seemed to be stable at around 20–30%. The prevalence of symptomatic LSS was higher in females than in males, especially in the 70–84 age group.

### 3.2. The Relationship between the Presence of Symptomatic LSS and Quality of Life (Qol)

First, a univariate analysis was performed to assess the relationship between each symptomatic LSS or comorbid conditions and quality of life (QoL) (eight physical/mental domains and two summary score of SF-36) (Table 3). QoL was remarkably different between the subjects with positive and negative symptomatic LSS. On the other hand, comorbid conditions did not always influence the QoL of the subjects.

Next, the multiple logistic regression analysis revealed that the presence of symptomatic LSS significantly negatively affected nine of ten QoL measurements except for mental component summary (MCS) (Table 4). People with symptomatic LSS had a significant 1.755-fold increase in their odds (95% confidence interval; CI: 1.262-2.441) of lower QoL than the national norm in physical functioning (PF), as well as in role-physical (RP): 1.789 (1.284–2.492), bodily pain (BP); 2.544 (1.806–3.585), general health perception (GH); 1.917 (1.466–2.507), vitality (VT); 2.139 (1.638–2.976), social functioning (SF); 1.547 (1.109–2.158), role-emotional (RE); 1.963 (1.411–2.731), mental health (MH); 1.559 (1.199–2.027); and 1.669 (1.190–2.341) in PCS compared to people without LSS. Although osteoarthritis of the hip (hip OA) and the knee (knee OA) and cardiovascular disease also showed a negative influence on some QoL measurements, the number of negatively affected QoL measures ranged from one to six of the ten factors (eight physical/mental domains and two summary scores of SF-36). With regard to the number of QoL measurements, LSS seemed to have the most potent negative influence on QoL compared to the comorbid conditions.

## 4. Discussion

Musculoskeletal disorders, such as lumbar spinal stenosis (LSS), are not directly related to mortality; however, they do have a strong influence on the quality of life (QoL) of patients [14–21]. The QoL is evaluated by condition-specific health status and general health-related quality of life (HR-QoL). The present study used the Medical Outcomes Study 36-Item Short Form Health Survey (MOS SF-36) for evaluating HR-QoL, that showed a strong negative impact of LSS on HR-QoL in the community setting. In particular, LSS was found to influence all eight physical/mental domains and physical component summary (PCS), but not mental component summary (MCS). In comparison with LSS in the clinic/hospital-based patients in previous studies, LSS in people in the community might have a stronger influence on HR-QoL [14–21]. These facts suggest that LSS could lead to serious health problems in the community setting.

There have been many epidemiological studies regarding lower back pain; however, there have been far fewer surveys of leg pain conducted in a community or population-based study [31–35]. Although most radiating leg pain and/or numbness in the elderly are thought to be caused by LSS, only a few reports on LSS in the community are available [8–10]. In the present study, a diagnostic support tool, LSS-DST, was used to define LSS [13]. LSS-DST was a questionnaire which showed good reliability and validity and was easy to use because it consisted of just 10 yes/no questions. This questionnaire was therefore highly suitable for an epidemiological study to survey the presence of LSS in a given population because of easy assessment, safety (no radiation exposure), and low cost (without imaging such as radiography and magnetic resonance imaging).

Previous studies have shown abnormal MRI findings such as disc degeneration, disc bulging, herniated disc, and/or a stenotic condition of the spinal canal to be common and gradually increase with age in asymptomatic subjects [36, 37]. These facts are widely accepted; however, there have been few reports on the prevalence of symptomatic LSS in the community. In the present study, the prevalence of leg symptoms caused by LSS gradually increased with age in the same way as preoperative patients in hospital [8, 20]. Many people live to an advanced age in Japan, which has now become an aging society. In 2005, when the present study was performed, 20.1% of the population was more than 65 years old. By 2025, it is estimated that more than 30% of the population will be over 65. Therefore it is increasingly necessary to ensure good health for all generations, especially the elderly, since they will comprise such a large proportion of the population. The results of the present study indicate that LSS might be one of the most serious health problems affecting active life in the elderly, because LSS has a high prevalence as well as a strong negative influence on QoL.

This study has several limitations. First, there was a lack of imaging and clinical evaluation for the definition of LSS. Regarding the questionnaire, LSS-DST was fairly effective; however, approximately 20% of LSS positive patients were suspected to be false positive. Additionally, there was no assessment of the severity of clinical symptoms or physical findings and no comparison between severity of LSS and QoL. Second, this was a cross-sectional study. Third, the research location was in a local and highly mountainous area, so the data may not extrapolate completely to the general Japanese population. Fourth, the reliability of knee and hip OA judgement between the physicians was not assessed. Finally, all of the participants in this study were volunteers, so there could be unintentional sample bias. For example, the prevalence of symptomatic LSS resulting from this study may differ from the true prevalence of LSS in Japan because of some deviation in the voluntary participants. In spite of these limitations, the present study might still have worth because this is the first study to show that LSS should be considered to be one of the most serious health problems in comparison with other comorbidities in the community-living elderly. In addition, further study is needed regarding preventability of LSS and possible application of our study results in the promotion of health in the community.

## 5. Conclusion

Symptomatic lumbar spinal stenosis (LSS) is common in the elderly. The presence of symptomatic LSS can have a negative effect on general health-related quality of life (HR-QoL), having a negative impact especially on physical rather than mental status. To maintain and improve quality of life (QoL) through the prevention and treatment of musculoskeletal diseases in the elderly, we believe that LSS is one of the most serious health problems that should be targeted because of its high prevalence and strong negative influence on QoL.

## Figures and Tables

**Table 1:** Age-specific participation rate of the local population (total, male/female).

Age	Population			Participant			*B*/*A* × 100 (%)
	Male	Female	M + F (*A*)	Male	Female	M + F (*B*)	
15–19	213	207	420	0	2	2	0.5
20–24	176	135	311	8	8	16	5.1
25–29	179	157	336	11	8	19	5.7
30–34	207	181	388	22	16	38	9.8
35–39	227	185	412	23	19	42	10.2
40–44	263	213	476	22	34	56	11.8
45–49	290	278	568	36	41	77	13.6
50–54	349	322	671	42	78	120	17.9
55–59	444	320	764	49	84	133	17.4
60–64	341	360	701	78	144	222	31.7
65–69	385	448	833	104	205	309	37.1
70–74	442	507	949	117	235	352	37.1
75–79	342	438	780	114	183	297	38.1
80–84	191	366	557	51	85	136	24.4
85–89	101	222	323	16	17	33	10.2
90–94	48	123	171	3	2	5	2.9

	4198	4462	8660	696	1166	1862	21.5

**Table 2:** Age-specific prevalence of lumbar spinal stenosis (LSS) (total, male/female).

Age	Prevalence of LSS (%)		*P* value
	Male	Female	
15–19	—	0	—
20–24	0	0	1.0
25–29	9.1	0	0.579
30–34	4.5	6.3	0.671
35–39	0	5.3	0.452
40–44	4.5	5.9	0.661
45–49	8.3	7.3	0.598
50–54	4.8	14.1	0.100
55–59	16	14.3	0.486
60–64	16.7	16.6	0.561
65–69	19.8	20.4	0.516
70–74	20.5	28.5	0.006
75–79	22.4	33.5	0.026
80–84	28	44.2	0.045
85–89	18.8	47.1	0.087
90–94	66.7	50	0.7

**Table 3:** Results of univariate analysis between lumbar spinal stensis (LSS) or the other seven comorbid conditions and quality of life (QoL).

	LSS		Knee OA		Hip OA		Hypertension		Cerebrovascular disease		Respiratory disease		Cardiovascular disease		Diabetic mellitus	
	+	−	+	−	+	−	+	−	+	−	+	−	+	−	+	−
PF	68.8	80.7^1^	71.1	81.8^1^	75.0	78.5	75.7	79.1^4^	60.2	78.1^6^	76.7	77.9	75.0	78.2	74.2	78.0
	48.2	50.9^4^	49.1	51.0^1^	48.7	50.4^5^	50.9	50.4	44.6	50.7^6^	52.6	50.6	51.2	50.5	49.7	50.6
RP	50.8	70.7^1^	53.6	72.7^1^	67.2	66.4	61.7	68.2^6^	51.6	66.0	25.0	65.9^7^	51.6	67.5^2^	59.7	66.0
	45.2	48.8^1^	45.8	49.1^1^	47.5	48.1	47.8	48.2	45.7	48.1	38.4	48.1^7^	45.7	48.4^5^	46.7	48.1
BP	55.9	71.1^1^	61.1	71.3^1^	64.0	68.2	67.2	68.8	64.6	68.2	55.3	68.2	65.2	68.5	63.2	68.4^7^
	45.0	50.1^1^	47.3	49.9^1^	46.7	49.1^7^	49.3	49.3	48.6	49.3	45.6	49.3	48.8	49.4	47.8	49.4
GH	51.9	64.5^1^	55.8	64.1^2^	54.5	62.1^2^	59.3	63.3^2^	48.1	62.1^3^	48.3	61.9^7^	51.9	62.9^1^	56.7	62.1^6^
	46.7	51.7^1^	48.5	51.4^1^	46.8	50.9^2^	50.0	51.2^6^	45.7	50.9^5^	45.4	50.8	47.2	51.2^1^	48.4	50.9^6^
VT	62.9	71.1^1^	66.5	71.1^1^	67.7	69.5	69.3	69.6	65.4	69.6	55.6	69.6	69.1	69.6	66.1	69.7
	49.7	52.9^1^	51.3	52.9^5^	51.1	52.3	52.4	52.3	50.5	52.4	46.8	52.4	52.5	52.4	51.1	52.4
SF	81.8	86.9^1^	84.7	86.4	84.2	85.9	84.4	86.8^7^	83.6	86.0	91.7	85.9	84.5	86.1	84.1	86.0
	49.8	51.3	51.0	51.0^7^	49.7	51.0	50.7	51.3	50.4	51.1	54.4	51.1	51.0	51.1	50.5	51.1
RE	57.9	74.7^1^	62.2	75.3^1^	71.0	71.2	65.0	73.5^4^	66.7	70.5	22.2	70.6^6^	59.3	71.8^4^	62.7	70.8
	46.0	49.3^6^	47.1	49.3	48.0	48.7	47.6	49.1^6^	47.8	48.6	37.1	48.6^7^	46.5	48.8^6^	46.6	48.7
MH	69.7	75.4^1^	72.5	74.7^6^	72.3	74.3	74.3	74.5	74.3	74.4	74.0	74.4	71.7	74.7^7^	72.0	74.5
	48.4	51.1^1^	49.9	50.1^7^	49.4	50.6	50.6	50.6	51.2	50.6	50.1	50.6	49.4	50.7^7^	49.6	50.7
PCS*	45.2	48.1^1^	45.4	48.4^1^	47.8	47.5	47.7	47.5	43.2	47.6^7^	38.0	47.6^7^	46.3	47.4^7^	46.3	47.6
MCS*	51.1	52.6	52.3	52.2	50.0	52.3^5^	52.3	52.4	53.2	52.3	56.9	52.3	51.9	52.4	52.3	52.4

Upper: average of original score (0−100).

Lower: average of standardized score (average = 50, one-standard deviation = 10).

*PCS, MCS: only standardized score is available.

^1^ *P* < 0.0001, ^2^ *P* < 0.0005, ^3^ *P* < 0.001, ^4^ *P* < 0.005, ^5^ *P* < 0.01, ^6^ *P* < 0.05, and  ^7^ *P* < 0.1.

PF: physical functioning; RP: role-physical; BP: bodily pain; GH: general health perception; VT: vitality; SF: social functioning; RE: role-emotional; MH: mental health; PCS: physical component summary; MCS: mental component summary.

**Table 4:** Results of multiple logistic regression analysis between lumbar spinal stenosis (LSS), the other seven comorbid conditions, body mass index (BMI), or smoking and quality of life (QoL).

	BMI	Smoking exp. (pack·year)	LSS	Knee OA	Hip OA	Hypertension	Cerebrovascular disease	Respiratory disease	Cardiovascular disease	Diabetic mellitus
PF										
Odds ratio	0.994	1.001	**1.755**	**1.608**	1.242	0.945	1.856	0.769	1.185	1.623
95% CI	0.951,	1.000	1.262,	1.197,	0.671,	0.705,	0.650,	0.064,	0.766,	0.842,
	1.039	1.001	2.441	2.160	2.299	1.266	5.298	9.263	1.832	3.130
RP										
Odds ratio	0.991	1.000	**1.789**	**1.717**	1.009	1.158	1.343	1.242 · 10^7^	1.369	0.943
95% CI	0.950	1.000	1.284,	1.287,	0.549,	0.875	0.459,	0.000	0.890,	0.487,
	1.034	1.001	2.492	2.291	1.853	1.534	3.928	—	2.104	1.828
BP										
Odds ratio	1.009	1.000	**2.544**	**1.408**	**2.364**	0.891	1.386	6.903 · 10^6^	1.132	1.745
95% CI	0.968,	1.000	1.806,	1.057,	1.212,	0.675,	0.455,	0.000	0.732,	0.879,
	1.051	1.001	3.585	1.876	4.612	1.177	4.227	—	1.752	3.464
GH										
Odds ratio	0.986	1.000	**1.917**	**1.331**	1.462	1.219	1.548	3.074	**1.734**	1.256
95% CI	0.954,	1.000	1.466,	1.056,	0.886,	0.977,	0.664,	0.599	1.200,	0.772,
	1.018	1.000	2.507	1.678	2.413	1.522	3.610	15.77	2.507	2.043
VT										
Odds ratio	1.023	1.000	**2.139**	1.239	1.048	0.834	1.596	0.852	1.013	1.519
95% CI	0.979,	1.000,	1.638,	0.918,	0.560,	0.619,	0.560,	0.071,	0.644,	0.790,
	1.070	1.000	2.976	1.673	1.962	1.123	4.548	10.226	1.594	2.920
SF										
Odds ratio	0.989	1.000	**1.547**	1.003	1.420	1.248	1.144	1.823/10^7^	0.987	0.798
95% CI	0.947,	1.000,	1.109,	0.743,	0.772,	0.936,	0.379,	0.000,	0.632,	0.395,
	1.033	1.001	2.158	1.353	2.610	1.665	3.303	—	1.540	1.611
RE										
Odds ratio	0.973	1.000	**1.963**	**1.375**	1.126	1.289	1.122	1.433 · 10^7^	1.402	1.125
95% CI	0.931,	1.000	1.411,	1.021,	0.613,	0.965,	0.390,	0.000	0.910,	0.571
	1.018	1.000	2.731	1.850	2.067	1.721	3.232	—	2.160	2.214
MH										
Odds ratio	**0.954**	1.000	**1.559**	0.992	1.205	0.993	0.614	0.817	1.269	0.907
95% CI	0.923,	0.999	1.199,	0.787,	0.746,	0.795,	0.266,	0.191,	0.891,	0.557,
	0.986	1.000	2.027	1.251	1.946	1.240	1.418	3.500	1.807	1.478
PCS										
Odds ratio	1.006	1.000	**1.669**	**1.898**	1.308	1.014	1.089	1.009 · 10^7^	**1.705**	0.833
95% CI	0.963,	1.000,	1.190,	1.413,	0.704,	0.761,	0.386,	0.000	1.090,	0.423,
	1.050	1.001	2.341	2.550	2.431	1.351	3.072	—	2.667	1.641
MCS										
Odds ratio	**0.950**	1.000	1.160	0.870	1.201	1.059	0.590	1.388/10^7^	0.896	1.388
95% CI	0.909,	0.999,	0.825,	0.643,	0.652,	0.794,	0.185,	0.000	0.569,	0.720,
	0.993	1.000	1.631	1.176	2.211	1.414	1.884	—	1.410	2.674
No. of positive subjects for HR-QoL	**2**	**0**	**0**	**0**	**0**	**0**	**0**	**0**	**0**	**0**
No. of negative subjects for HR-QoL	**0**	**0**	**9**	**6**	**1**	**0**	**0**	**0**	**2**	**0**
Overall prevalence (%)			**21.0**	**28.2**	**4.8**	**35.7**	**1.6**	**0.5**	**9.5**	**4.9**

PF: physical functioning; RP: role-physical; BP: bodily pain; GH: general health perception; VT: vitality; SF: social functioning; RE: role-emotional; MH: mental health; PCS: physical component summary; MCS: mental component summary.

No. of positive subjects for HR-QoL: number of positive influence on 10 aspects of SF-36.

No. of negative subjects for HR-QoL: number of negative influence on 10 aspects of SF-36.

## Appendix

### Diagnostic Support Tool for Lumbar Spinal Stenosis (LSS-DST) [13]

Q.1: Numbness and/or pain exist in the thighs down to the calves and shins.
  Yes/No
Q.2: Numbness and/or pain increase in intensity after walking for a while, but are relieved by taking a rest.
  Yes/No
Q.3: Standing for a while brings on numbness and/or pain in the thighs down to the calves and shins.
  Yes/No
Q.4: Numbness and/or pain reduced by bending forward.
  Yes/No
Q.5: Numbness is present in both legs.
  Yes/No
Q.6: Numbness is present in the soles of both feet.
  Yes/No
Q.7: Numbness arises around the buttocks.
  Yes/No
Q.8: Numbness is present, but pain is absent.
  Yes/No
Q.9: A burning sensation arises around the buttocks.
  Yes/No
Q.10: Walking nearly causes urination.
  Yes/No
